# A new HILIC-ICP-SF-MS method for the quantification of organo(fluoro)phosphates as decomposition products of lithium ion battery electrolytes

**DOI:** 10.1039/c9ra01291e

**Published:** 2019-04-11

**Authors:** Yannick Philipp Stenzel, Jonas Henschel, Martin Winter, Sascha Nowak

**Affiliations:** University of Münster, MEET Battery Research Center Corrensstraße 46 48149 Münster Germany sascha.nowak@uni-muenster.de +49 251 83 36032 +49 251 83 36735; Helmholtz-Institute Münster (HI MS), IEK-12, Forschungszentrum Jülich GmbH Corrensstraße 46 48149 Münster Germany

## Abstract

The lithium ion battery (LIB) is the most popular choice for powering consumer electronics, grid storage and electric vehicles. Decomposition reactions in LIBs, leading to so-called aging, are the main reason for loss of capacity and power and will affect LIB safety. Organo(fluoro)phosphates (O(F)Ps) as decomposition products of LIB electrolytes have been identified in several studies in the literature but quantitative data of O(F)Ps in LIBs are only scarcely available. In terms of toxicity, this substance class is highly relevant as it shows structural similarities to chemical warfare agents. Thus, approaches that can deliver quantitative data are in need. In this study, acidic O(F)Ps were quantified with an inductively coupled plasma-sector field-mass spectrometer (ICP-SF-MS) after separation of species with hydrophilic interaction liquid chromatography (HILIC). The formation of OFPs exceeds the amount of non-fluorine containing OPs by a factor of up to 15. A total of 16 different O(F)P compounds could successfully be quantified. Organic mass spectrometry was used for the assignment of quantitative data.

## Abstract 2

The lithium ion battery (LIB) is the most popular choice for powering consumer electronics, grid storage and electric vehicles.

## Graphical abstract

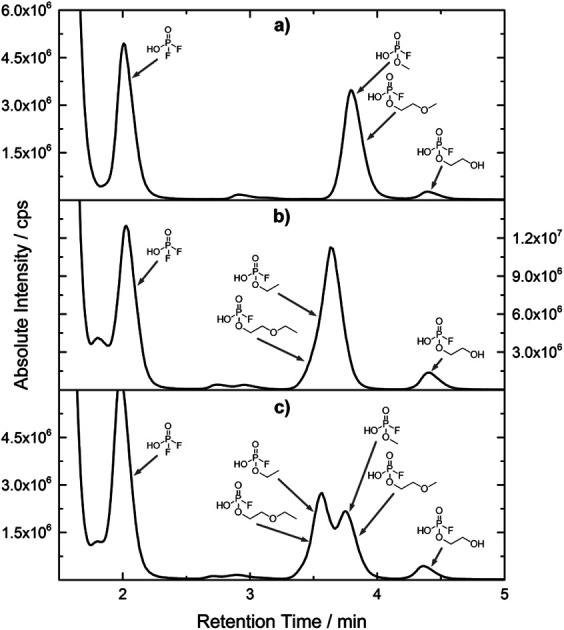

## 1. Introduction

The lithium ion battery (LIB) has become the preferential choice of energy supply in consumer electronics, grid storage and electric vehicles since its introduction almost three decades ago.^1–5^ The possibility of fast energy storage with high energy content and adequate safety properties means this technology is widely distributed all over the globe.^1,2,4^ State-of-the-art LIBs comprise a carbonaceous negative electrode and (transition) metal (Ni, Co, Mn, Al) oxides or phosphates as the positive electrode.^6–9^ A separator which is soaked with electrolyte separates the two electrodes.^10,11^ Although the electrolyte is referred to as ‘inactive material’, it is considered a key part of the LIB.^12,13^ It comprises a conducting salt (lithium hexafluorophosphate, LiPF_6_) which is dissolved in a mixture of both linear and cyclic carbonates.^14–16^ This formulation allows adequate salt concentrations and good ion transport properties in the desired voltage and temperature range.^17^ Although this electrolyte system is deemed state-of-the-art, several degradation effects (contributing to aging) have been reported in the literature.^18,19^ The formation of so-called solid electrolyte interphases (SEI) and cathode electrolyte interphases (CEI) is considered essential for the functionality of LIBs and thus a desired aging result.^20–23^ But loss of capacity and power as well as formation of disruptive aging products impairs the battery life and can deteriorate the safety properties.^24–27^ The decomposition of the conducting salt was intensely studied.^28–42^ As trace moisture is always present in LIBs, the moisture-sensitivity of LiPF_6_ initiates a decomposition cascade leading to the formation of several organo(fluoro)phosphate (O(F)P) compounds. Due to their structural similarity to chemical warfare agents (CWAs) this compound class is supposedly highly toxic due to an irreversible inhibition effect on the acetylcholinesterase (AChE) of the mammalian central nervous system leading to paralysis of the cardiac action and respiration eventually resulting in exitus.^43–45^ In the LIB context, this compound class was targeted in several studies but quantitative analysis of O(F)Ps is only scarcely available in literature.^34,46–48^ Due to lack of standard materials and registrations regarding synthesis, hyphenation of chromatographic techniques to elemental mass spectrometers (MS) was performed to obtain quantitative results.^33,35^

In this study, hyphenation of hydrophilic interaction liquid chromatography (HILIC) to elemental MS is evaluated for quantification of acidic O(F)Ps. The general separation mechanism of the HILIC is highly headgroup-dependent.^49^ Hydrophilic partitioning of the analytes takes place in an aqueous-rich layer on the surface of the stationary phase and the bulk of the mobile phase. There, polar functional groups of the analyte form hydrogen bonds with the stationary phase resulting in retention, also, electrostatic interaction between ionized functional groups and the stationary phase can lead to retention behavior of the sample. Changing the eluent concentration during a measurement leads to a different spray efficiency in the spray chamber when hyphenated to a plasma-based detector and thus to misleading intensities of analytes regarding quantification. Therefore, an isocratic approach was performed since it was assumed to be sufficient to separate groups of O(F)Ps. In this setup, the composition of the mobile phase does not change so the nebulization efficiency stays constant over the whole analysis time and influences on the plasma itself are minimized.

In this work, a methodical approach was used to evaluate the hyphenation technique with the aim to separate the acidic decomposition products. Both elemental and molecular mass spectrometers were used to identify and quantify the species of interest. Thermal aging of the sample was used to generate a large bandwidth of compounds to evaluate the separation and quantification behavior also in view of real sample analysis with respect to sample complexity as reported before in literature.^50^ Eventually, quantitative and qualitative data are interrelated with previously obtained results from other hyphenation techniques.

## 2. Experimental

### 2.1 Hydrophilic interaction liquid chromatography-inductively coupled plasma-sector field-mass spectrometry

For hydrophilic interaction liquid chromatography-inductively coupled plasma-sector field-mass spectrometry (HILIC-ICP-SF-MS) investigations a Prominence UFLC (Shimadzu) system was used with a strong anion exchange column (Hypersil GOLD SAX, 200 × 2.1 mm, 1.9 μm; Thermo Fisher Scientific) in HILIC mode. Acetonitrile (LC-MS Grade; Fisher Scientific) was used for dilution of samples (1 : 20) and eluent. Ammonium formiate (LC-MS Grade; Fisher Scientific) was used as eluent. The LC system was hyphenated to an ELEMENT XR™ (Thermo Fisher Scientific) using a cooled spray chamber (−5 °C), an inlet tube with a diameter of 1 mm. Further parameters can be obtained from Table 1. Quantification of compounds was performed with external calibration (*R*^2^ = 0.999); linearity was given between 1 ppm (with regard to the phosphorus atom); and 500 ppm using trimethyl phosphate (TMP; ≥99%, Merck) as calibration standard. The limit of detection was 319 ppb and the limit of quantification was 1063 ppb evaluated with the 3-sigma and 10-sigma criterion, respectively.

**Table 1:** Experimental parameters of the ICP-SF-MS and LC system

Instrumental parameter	Value
**ICP-SF-MS**	
Instrument	ELEMENT XR™
Forward power	1250 W (with shielded torch)
Cooling gas flow rate	16.0 L min^−1^
Auxiliary gas flow rate	0.9 L min^−1^
Sample gas flow rate	0.580 L min^−1^
Sampling/skimmer cones	Platinum
Isotopes monitored	Medium resolution ^31^P
Additional gas flow rate	O_2_ 55 mL min^−1^

**LC**	
Instrument	Prominence UFLC
Binary pumps	LC-20AD
Degasser	DGU-20A_3_
Autosampler	SIL-20AC
Column oven	CTO-20AC
Eluent	20 mM ammonium formiate, 87% ACN isocratic
Flow rate	0.35 mL min^−1^
Injection volume	1 μL
Oven temperature	40 °C
Column	Hypersil GOLD SAX 200 × 2.1 mm, 1.9 μm

### 2.2 Hydrophilic interaction liquid chromatography-ion trap-time of flight-mass spectrometry

For identification using HILIC hyphenation, an LCMS-IT-ToF™ system (Shimadzu) was used. Ionization was performed in ESI(−) mode at −4.5 kV. The ion trap operated in automatic MS^2^ mode with an ion accumulation time of 10 ms in MS^1^ and 60 ms in MS^2^, leading to a loop time of 280 ms. The mass range was set to *m*/*z* 80–250. The collision induced dissociation energy and gas flow were set to 30%. For further structure elucidation and confirmation purposes close to the limit of detection, manual MS^2^ and varied ion trap parameters were used. Instrument parameters with regard to column, mobile phase, injection volume, and oven temperature were adjusted analogously to HILIC-ICP-SF-MS hyphenation.

### 2.3 Thermal aging of electrolyte

The investigated electrolytes (BASF, Selectilyte™) consisted of 1 M LiPF_6_ in a 1 : 1 (w/w) mixture of ethylene carbonate (EC) and either dimethyl carbonate (DMC), ethyl methyl carbonate (EMC) or diethyl carbonate (DEC). For simplification reasons the electrolytes are further referred to as EL_DMC_, EL_DEC_ and EL_EMC_. Thermal aging was conducted *via* storage of electrolyte in aluminum screw cap vessels at 80 °C for three weeks. The container was opened in a dry room (H_2_O < 10 ppm).

## 3. Results and discussion

The chromatographic separation technique of HILIC can provide head group specific separation of species which is favorable when investigating OPs and OFPs. Although the report in literature indicated both substance classes as highly toxic,^51^ the general mechanism of action with the AChE suggests a higher toxicity of OFPs as fluorine constitutes a good leaving group.^52^ Isocratic separation results into a tradeoff regarding chromatographic peak shape and resolution, and single analysis time. But, it has the advantage of a consistent eluent composition, which is convenient with respect to plasma-based detection and other detection systems with a dependence of the mobile phase composition with respect to the baseline. Changing the eluent concentration during a measurement using gradient separation leads to a different spray efficiency in the spray chamber and thus to different intensities of analytes. This can be overcome with an orthogonal pump system that is connected after the separation column to maintain the mobile phase composition prior to the spray chamber (counter gradient). Another possibility is to add a known amount of the analyte of interest to both eluent containers and subsequently monitor a run without the introduction of a sample a baseline is produced that delivers a correction factor at every time of separation. Both approaches were evaluated, but no satisfactory results could be obtained. The use of a counter gradient results in two additional pumps in the setup and a dilution of the sample. The implementation of a correction factor showed a bad reproducibility of samples with a known concentration of analyte. Eventually, an isocratic approach was performed since it was assumed to be sufficient to separate groups of O(F)Ps. In this setup the composition of the mobile phase does not change so the nebulizer efficiency stays constant over the whole analysis time. The use of an ICP-SF-MS as detection unit in medium resolution for phosphorus provides a reliable detection of the ^31^P isotope without any interferences. For quantification of generated species, three different commercially available electrolyte compositions were used: EL_DMC_, EL_DEC_ and EL_EMC_. The respective electrolytes were thermally treated (=aged) to artificially generate a bandwidth of phosphorous decomposition products. Table 2 shows the structures of the chromatograms of the different electrolytes. Compounds are depicted depending on their retention time. If coelution was present or chromatographic resolution was not sufficient for peak separation, letters are attributed to the structures of the respective peak group. For that, organic mass spectrometry was used for structural elucidation. In ensuing discussion of results, the reader is referred to Table 2 for assignment of compounds.

**Table 2:** Assignment of the decomposition products including the structure of the species, their structure and relative quantities in the respective electrolytes in the sample with respect to phosphorus

#	Structure	Retention time	EL_DMC_ in ppm	EL_DEC_ in ppm	EL_EMC_ in ppm
1	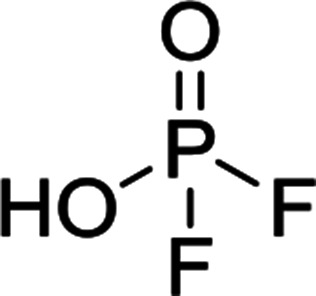	2.0 min	2142.2	4526.8	2888.3
2	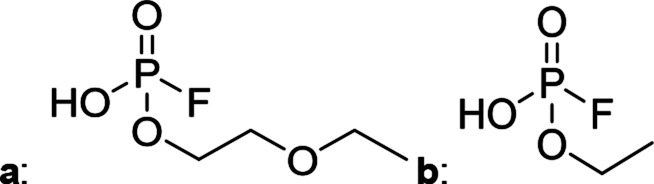	3.6 min	—	a + b 6953.3	2a + 2b + 3a + 3b 2720.2
3	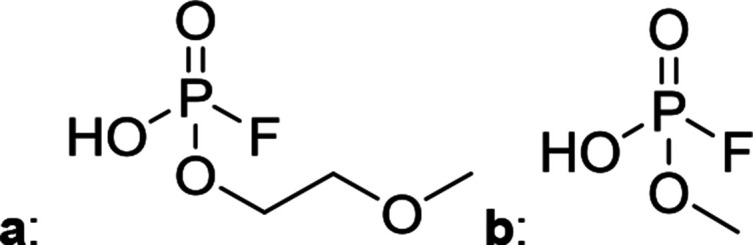	3.7 min	a + b 1893.1	—	
4	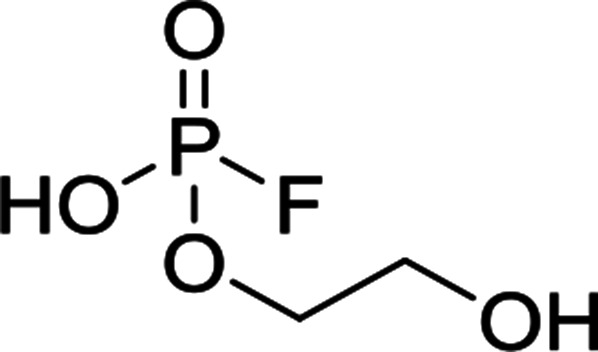	4.4 min	101.5	644.2	235.2
5	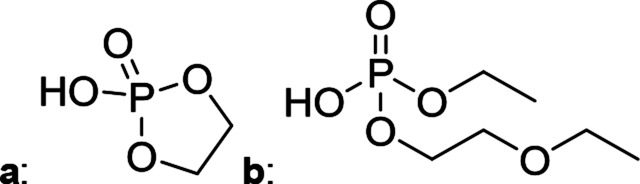	9.8 min	a 211.8	a + b 541.4	a + b 249.5
6	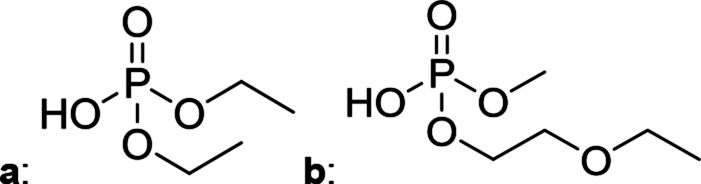	10.4 min	—	a 199.1	a + b 33.6
7	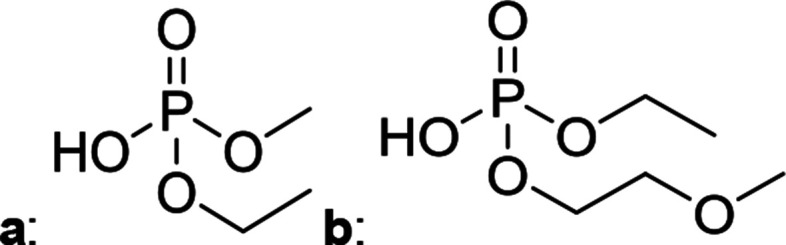	11.4 min	—	—	a + b 52.9
8	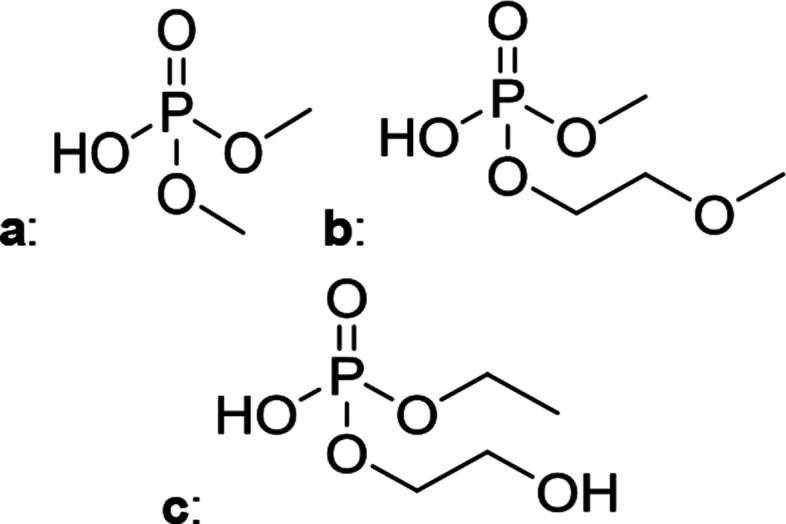	12.3 min	a + b 86.7	c 50.0	a + b + c 33.2
9	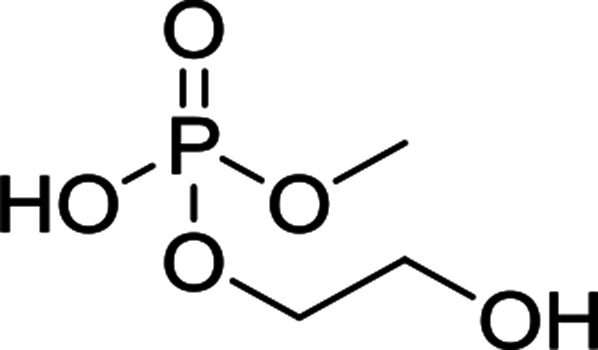	13.7 min	23.6	—	16.2

**Fig. 1:**
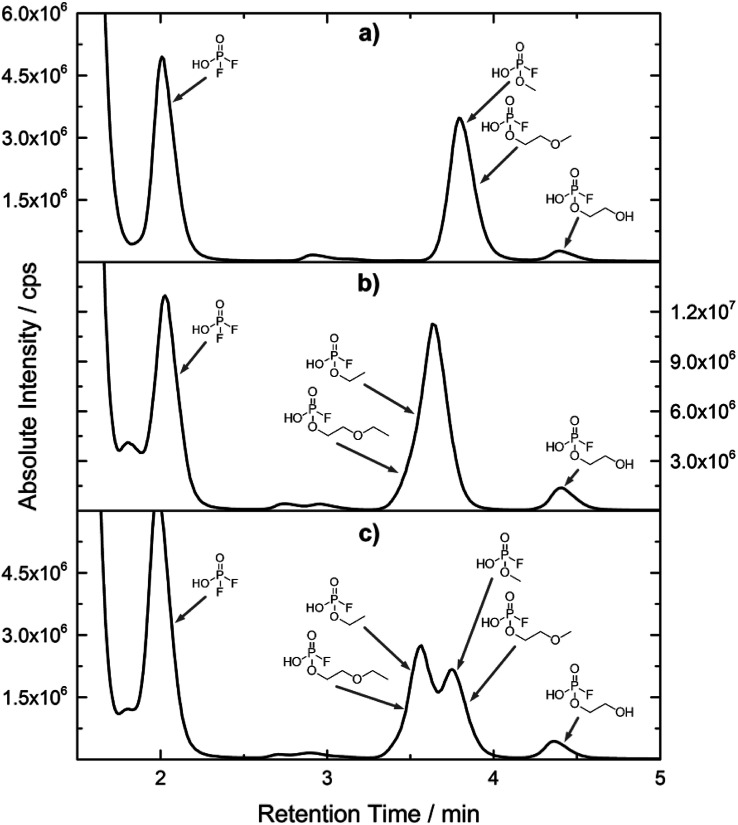
HILIC-ICP-SF-MS chromatogram of three thermally aged electrolytes. (a) EL_DMC_ (b) EL_DEC_ (c) EL_EMC_. The retention area of OFPs is depicted. Structures that were obtained from HILIC-IT-ToF experiments are assigned to signals of the chromatogram. The ^31^P trace is shown in medium resolution.

Fig. 1 shows the stacked chromatogram for the thermally aged electrolytes for (a) EL_DMC_ (b) EL_DEC_ and (c) EL_EMC_. The excerpt of the chromatogram shows early retention times where OFPs (1–4) were identified in HILIC-IT-ToF experiments. The peaks were assigned to the respective structures including side-chain variation in agreement with retention behavior.

In Table 2, quantities are given for structures in the respective electrolyte; compounds are depicted with progressive retention times of the respective chromatograms. Difluorophosphate (1) and ethoxy fluorophosphate (4) were present for every electrolyte composition. Compliant with the decomposition route, OFPs were detected depending on the electrolyte composition that was investigated. Two OFPs could be identified with organic mass spectrometry and detected with the plasma-based hyphenation technique (as one peak) for the respective electrolytes EL_DMC_ (3a + b) and EL_DEC_ (2a + b). For the electrolyte comprising an asymmetric linear carbonate (EL_EMC_), all four aging products (2a + b, 3a + b) could be detected.

**Fig. 2:**
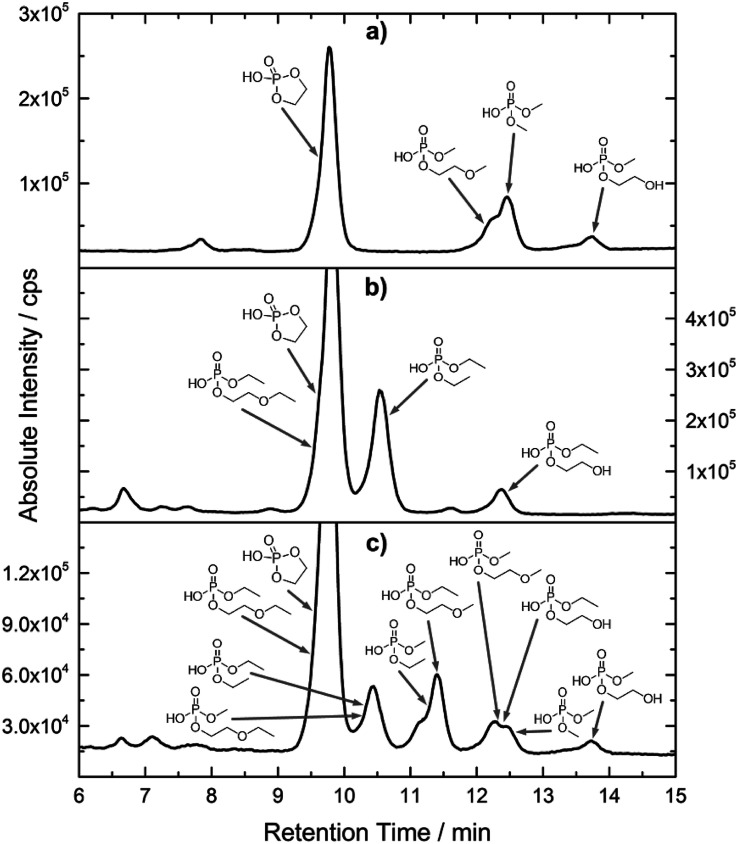
HILIC-ICP-SF-MS chromatogram of three thermally aged electrolytes. (a) EL_DMC_ (b) EL_DEC_ (c) EL_EMC_. The retention area of OPs is depicted. Structures that were obtained from HILIC-IT-ToF experiments are assigned to signals of the chromatogram. The ^31^P trace is shown in medium resolution.

Fig. 2 shows the subsequent part of the chromatogram of the investigated electrolytes (a) EL_DMC_, (b) EL_DEC_ and (c) EL_EMC_. In this chromatographic section, OPs were detected. Ethylene phosphate (5a) was present for all electrolytes as a reaction product of EC and the conducting salt. Generally, the decomposition products could also be attributed to the type of linear carbonate that was used in the respective electrolyte. Comparable decomposition products as at early retention times were present for the symmetric linear carbonate-containing electrolytes. The identified decomposition products contained next to the hydroxyl-group, methoxy and ethoxy sidechains, respectively. Depending on the electrolyte, a second methoxy (8a) and ethoxy (6a) sidechain were present. Additionally, ethoxymethyl (8b) and ethoxyethyl (5b) are visible in the chromatogram (Fig. 2a and b); a structure containing a hydroxyethyl sidechain (EL_DMC_: 9, EL_DEC_: 8c) is present for both electrolyte composition. This mixture of sidechains was also obtainable for the OFPs at earlier retention times (Fig. 1). The identified OPs for EL_EMC_ did not only comprise decomposition products of EL_DMC_ and EL_DEC_ but also showed intermixtures of the respective ethoxy and methoxy functional group (ending) (6b, 7a, 7b) in Fig. 2c. Quantification of decomposition products that contain terminal hydroxyl-groups was shown for the first time.

In general, identification of compounds was possible using the advantages of the molecular MS due to the high resolution setup and the possibility to perform sequential MS^*n*^ as reported in literature.^53^ The depicted chromatograms in Fig. 1 and 2 show the ^31^P trace in medium resolution. The retention behavior of compounds is reasonable due to highly headgroup dependence in HILIC separation (containing fluorine: two > one > zero). Side chains that increased the hydrophobicity consequently led to reduced retention times. Chromatographic separation could not be achieved for all compounds, but quantification of structural similar compounds was performed using external calibration with a trimethyl phosphate standard. The need for a two-dimensional setup as it was reported before could be overcome using this developed HILIC separation method.^35^ Quantitative results differed significantly from each other with regard to total quantities of phosphorous decomposition products in the sample: 4.4‰ (EL_DMC_), 12.9‰ (EL_DEC_) and 6.4‰ (EL_EMC_); quantities were always given with respect to the phosphorus atom. Since molecular contributions to the total quantities could not be given since coelution and (for some peaks) poor chromatographic resolution was present, total results have to be interpreted carefully. In addition, it was reported that different moisture content of the electrolytes influenced the decomposition route of LiPF_6_,^35^ thus comparability between thermally aged electrolytes is challenging. Some signals of the chromatogram could not be assigned to phosphorous decomposition products, which extends the uncertainty in terms of total quantities. A clear trend is observable when investigating the factor between the quantities of OPs and OFPs. The quantities of the identified OFPs exceed the OPs by a factor of 13 (EL_DMC_) to 15 (EL_DEC_ and EL_EMC_). This excess of compounds containing fluorine is compliant with the general decomposition route and previous investigations of non-acidic O(F)Ps in reports in literature.^47^

## 4. Conclusions

Investigations on acidic O(F)Ps in thermally aged electrolytes were performed in this work. Analogously to GC investigations with both organic and inorganic mass spectrometric hyphenation techniques,^33^ HILIC was used for separation of acidic species. LC separation was performed in isocratic elution mode which was preferred for hyphenation to plasma-based techniques. Individual decomposition products that were consistent with the electrolyte formulation (especially with respect to the change of the linear carbonate) that was used, could be described; this also included longer sidechains and their respective functional groups. Eventually, identification and quantification of sixteen O(F)Ps were successful. In this regard, species with the highest quantities could be identified and quantified. Although complete species-separated quantification was not possible for all compounds, headgroup-dependent separation could be achieved. The OFPs were shown in quantitatively more relevant amounts exceeding the quantities of OPs not containing fluorine by a factor of up to 15. The practical impact and importance of quantitative findings also regarding potential toxicological and environmental consequences will be targeted in future studies.

## Conflicts of interest

There are no conflicts to declare.

## Supplementary Material
